# Enhancing Therapeutic Efficacy of Curcumin: Advances in Delivery Systems and Clinical Applications

**DOI:** 10.3390/gels9080596

**Published:** 2023-07-25

**Authors:** Hossein Omidian, Renae L. Wilson, Sumana Dey Chowdhury

**Affiliations:** Barry and Judy Silverman College of Pharmacy, Nova Southeastern University, Fort Lauderdale, FL 33328, USA; rw1273@mynsu.nova.edu (R.L.W.); sd2236@mynsu.nova.edu (S.D.C.)

**Keywords:** curcumin gel formulations, transdermal delivery, periodontal therapy, ocular inserts, gastrointestinal bioavailability

## Abstract

Curcumin, a potent active compound found in turmeric and Curcuma xanthorrhiza oil, possesses a wide range of therapeutic properties, including antibacterial, anti-inflammatory, antioxidant, and wound healing activities. However, its clinical effectiveness is hindered by its low bioavailability and rapid elimination from the body. To overcome these limitations, researchers have explored innovative delivery systems for curcumin. Some promising approaches include solid lipid nanoparticles, nanomicelle gels, and transdermal formulations for topical drug delivery. In the field of dentistry, curcumin gels have shown effectiveness against oral disorders and periodontal diseases. Moreover, Pickering emulsions and floating in situ gelling systems have been developed to target gastrointestinal health. Furthermore, curcumin-based systems have demonstrated potential in wound healing and ocular medicine. In addition to its therapeutic applications, curcumin also finds use as a food dye, contraception aid, corrosion-resistant coating, and environmentally friendly stain. This paper primarily focuses on the development of gel compositions of curcumin to address the challenges associated with its clinical use.

## 1. Introduction

**Scheme 1:**
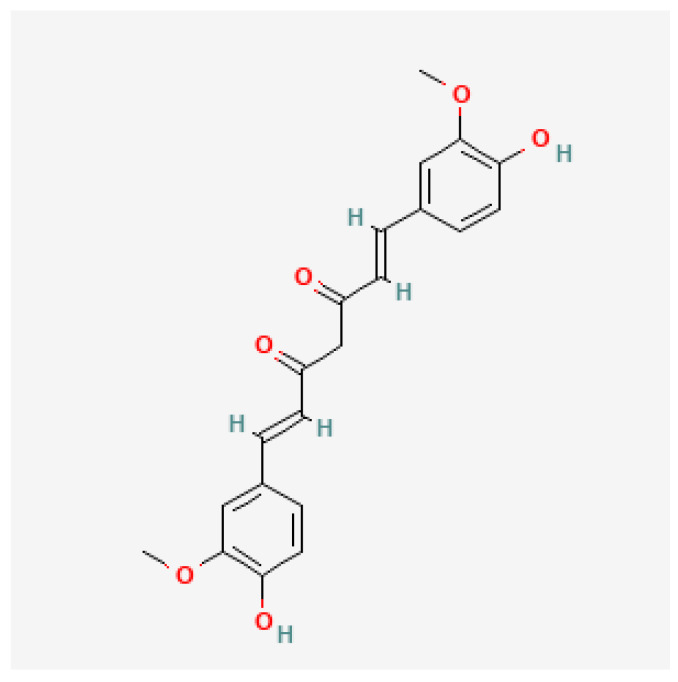
Chemical structure of curcumin, diferuloylmethane [1].

Curcumin, also known as diferuloylmethane (Scheme 1), is an active component in the golden spice turmeric (Curcuma longa) and in Curcuma xanthorrhiza oil [1]. It is a highly pleiotropic molecule that exhibits antibacterial, anti-inflammatory, hypoglycemic, antioxidant, wound healing, and antimicrobial activities. Due to these properties, curcumin has been investigated for the treatment and supportive care of clinical conditions including proteinuria, breast cancer, multiple myeloma, depression, and non-small-cell lung cancer (NSCLC) [2]. Despite proven efficacy against numerous experimental models, poor bioavailability due to poor absorption, rapid metabolism, and rapid systemic elimination have been shown to limit the therapeutic efficacy of curcumin [2,3]. To overcome these challenges, researchers have focused on developing innovative curcumin delivery systems and formulations that enhance stability, bioavailability, and targeted delivery [4,5].

The development of curcumin formulations has been a subject of intense investigation encompassing a wide array of strategies and approaches. Solid lipid nanoparticles (SLNs) loaded with curcumin incorporated into a thermoresponsive gel have shown promise for Alzheimer’s disease treatment [6]. Similarly, nanoparticle-incorporated hyaluronic acid gel has demonstrated a preventive effect against peritoneal adhesions [4], and mixed hydrogels of whey protein aggregates and k-carrageenan have shown the ability to protect and deliver curcumin to the colon [5]. A curcumin phytosomal soft gel formulation has also been developed to enhance bioavailability and dissolution characteristics [7]. Surface modification of MIL-100(Fe) with carboxymethyl cellulose enables slow release of curcumin [8]. Starch-based emulsion gel beads and curcumin–pectin calcium gel beads have shown potential for targeted delivery and functional food applications [9,10].

Liposomal solid gels and chitosan-based hydrogels have been investigated to address curcumin’s solubility and bioavailability issues [11,12]. Ophthalmic in situ gels, optical pH sensors, cryogel encapsulation, and film-forming hydrogels have been developed as efficient drug delivery systems [13,14,15,16]. Curcumin has shown promise in malarial parasite management systems [17] and demonstrated improved anti-hepatitis-C-virus delivery in liposomal solid gels [18].

In the field of topical applications, various curcumin delivery systems have been developed to overcome its poor bioavailability and limited skin permeability. Proniosomal gels, nanomicelle gels, and transdermal gel formulations have been explored for topical drug delivery [19,20,21]. Nanostructured lipid carriers, organogels, mucoadhesive gels, and nanosponge-based gels offer alternative approaches [22,23,24,25]. Transdermal delivery systems such as ethosomal gels, molecular inclusion complexes, transfersomes, and gel microemulsions have shown promise in enhancing curcumin’s skin permeation [26,27,28,29].

In dentistry, curcumin gel formulations have demonstrated therapeutic benefits for various oral conditions. Studies have explored curcumin gel for treating minor recurrent aphthous stomatitis (RAS) and premalignant oral disorders (oral leukoplakia) [30,31]. Curcumin’s antimicrobial capacity has been investigated in root canals [32], and it has shown antiplaque and anti-inflammatory effects when used in gingivitis management [33]. Furthermore, curcumin gel has been studied in the context of periodontal disease management, including management of chronic periodontitis [34], periodontal pockets [35], and azole-resistant oral candidiasis [36].

Curcumin-based delivery systems have also shown potential in managing conditions in oral health. In the case of oral submucous fibrosis (OSMF), curcumin gel and buccal mucoadhesive patches have demonstrated efficacy [37]. Combination therapies involving curcumin gel, triamcinolone–hyaluronidase gel, aloe vera gel, and oral physiotherapy have been explored to enhance OSMF management [38,39].

Gastrointestinal health has also been a focus of curcumin delivery system research. Curcumin-loaded Pickering emulsions, emulsion gels, and oil-filled aerogels offer potential solutions for enhancing curcumin’s bioavailability and stability during digestion [40,41]. Gastroretentive floating in situ gelling systems have been formulated for gastric ulcer treatment, targeting Helicobacter-pylori-associated ulcers [42,43]. Moreover, ultra-high-pressure technology has been utilized to fabricate emulsion gels with unique physical characteristics and controlled release properties [44].

In wound healing, curcumin-based delivery systems have shown promise in overcoming solubility and bioavailability challenges. Nanogel formulations, such as silver–curcumin nanoparticles, have been evaluated for burn healing efficacy and safety [45]. Self-assembled nanogels, gelatin-based gels, and self-microemulsifying gels offer innovative solutions for curcumin delivery and wound healing [46,47,48]. Furthermore, nanocrystals and sol-gel coatings have been developed to enhance curcumin’s solubility, stability, and wound healing potential [49,50]. Comparisons with conventional treatments, such as triamcinolone oral paste, have further highlighted the potential of curcumin gel in wound healing [51].

In ocular medicine, innovative delivery systems have been developed based on curcumin’s therapeutic potential. Ocular inserts and in situ gelling systems have been developed for long-acting ocular delivery of curcumin, addressing its poor solubility and short ocular residence time [52,53]. These advancements offer promise for the effective treatment of ocular diseases using curcumin-based therapies.

Moreover, other than therapeutic applications, curcumin has been investigated for its use as a natural dye alternative in food products [54], a component in vaginal gels for contraception [55], a corrosion-resistant coating for mild steel surfaces [56], and an environmentally friendly protein stain [57]. Curcumin-based delivery systems have also been explored for controlled curcumin delivery in food matrices [58,59], efficient drug delivery [60,61], and intranasal neurological applications [62].

This study explores the wide-ranging potential of curcumin as a therapeutic agent in various fields. It aims to review and analyze innovative curcumin delivery systems that address challenges such as poor solubility and limited bioavailability. By enhancing our understanding of these delivery methods, the study seeks to pave the way for more effective treatments and improved patient outcomes in different medical areas.

## 2. Curcumin Delivery Systems

To overcome the poor pharmacokinetic properties of curcumin, researchers have delved into developing various delivery systems. One approach investigated the development of a microemulsion-based in situ ion-sensitive gelling system for the intranasal administration of curcumin. This novel system was designed to improve the absorption and brain targeting of curcumin through nasal delivery. The microemulsion was optimized using a simple lattice design, and its physicochemical properties were thoroughly investigated. Notably, histological section studies demonstrated the safety of this system for nasal mucosa. The pharmacokinetic results showed a significant improvement in the absolute bioavailability of curcumin, reaching 55.82% by intranasal administration, and a remarkable brain-targeting index (BTI) of 6.50. This enhanced brain targeting was attributed to direct nose-to-brain drug transport, making the microemulsion-based in situ ion-sensitive gelling system an effective and safe vehicle for the intranasal delivery of curcumin [62].

Another approach was the development of a thermoresponsive ophthalmic in situ gel containing curcumin-loaded albumin nanoparticles (Cur-BSA-NPs-Gel) for ocular drug delivery. Albumin nanoparticles were prepared via a desolvation method, and the gels were prepared using a cold method. The formulation was optimized to undergo a sol–gel transition at a specific temperature, allowing for easy administration and improved bioavailability. In vitro and in vivo studies in rabbits demonstrated the safety and increased bioavailability of curcumin in the aqueous humor with this optimized formulation. The Cur-BSA-NPs-Gel achieved superior sustained release effects, and the incorporation of albumin nanoparticles had minimal impact on the gel structure, highlighting its potential for ocular application [13].

Furthermore, researchers explored a nanogel combining cationic nanostructured lipid carriers (CNLC) and a thermosensitive gelling agent to enhance the preocular retention and ocular permeation capacity of curcumin. The thermosensitive ophthalmic in situ nanogel of CUR-CNLC (CUR-CNLC-GEL) demonstrated a solution–gel transition temperature suitable for ocular administration. The CUR-CNLC-GEL exhibited zero-order release kinetics and significantly improved curcumin permeation compared to a curcumin solution. The formulation’s controlled release properties indicate its potential as a promising option for enhancing bioavailability in the aqueous humor and improving corneal permeation and retention capacity [63].

Moreover, researchers explored the use of copolymeric micelles to modify the pharmacokinetics and tissue distribution of curcumin. A synthesized poly(D,L-lactide-co-glycolide)-b-poly(ethylene glycol)-b-poly(D,L-lactide-co-glycolide) (PLGA-PEG-PLGA) copolymer was used to prepare CUR-loaded micelles. These micelles demonstrated improved pharmacokinetic parameters, including increased plasma AUC(0–∞), t1/2α, t1/2β, and MRT compared to a CUR solution. In vivo biodistribution studies in mice showed reduced drug uptake by the liver and spleen, while drug distribution was enhanced in the lung and brain. These results highlight the potential of PLGA-PEG-PLGA micelles as an effective carrier for curcumin, offering improved tissue distribution and bioavailability [64].

**Figure 1:**
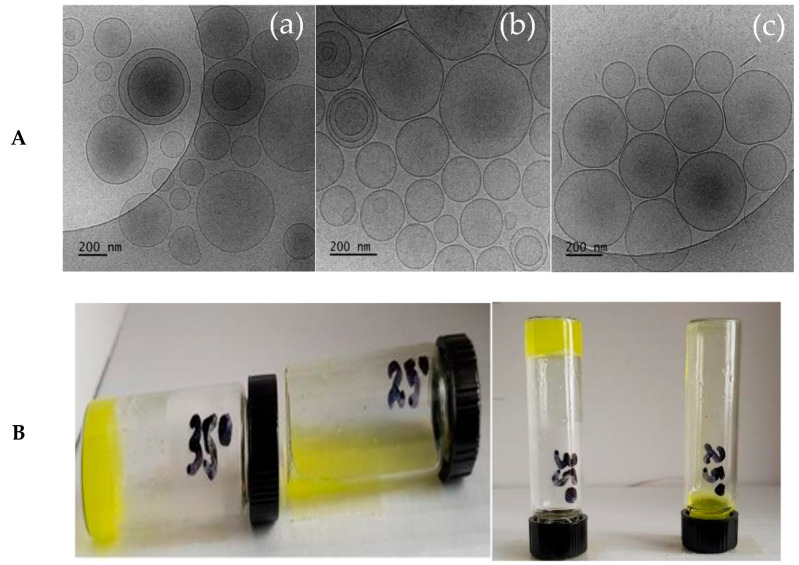

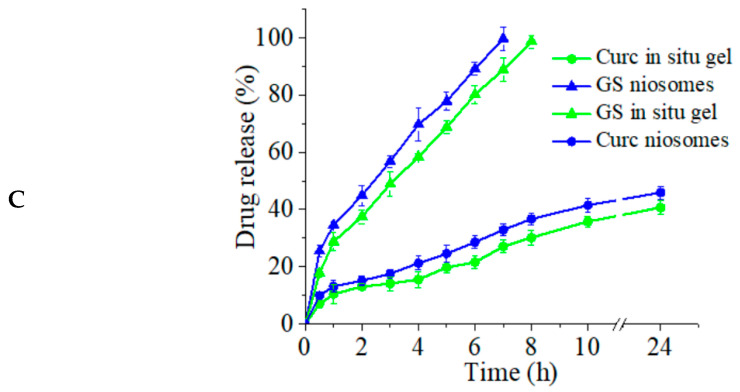
(**A**) Cryo-TEM images of niosomes (a) empty, (b) loaded with curcumin, and (c) loaded with curcumin and gentamicin (Curc/GS co-loaded); (**B**) Curc-GS niosomal thermosensitive in situ gel at 35 °C and at ambient temperature; (**C**) in vitro release profiles of curcumin and gentamicin sulfate from selected Curc/GS co-loaded niosomes and from hybrid niosomal in situ gel [65].

Other innovative strategies have been explored to enhance the properties of curcumin and its drug delivery systems. Amalia et al. modified MIL-100(Fe) with carboxymethyl cellulose (CMC) to achieve pH-responsive drug delivery potential and suppressed release of curcumin [8]. Furthermore, Bu et al. developed starch-based emulsion gel beads capable of dual loading proanthocyanidin and curcumin which showed tolerance to gastric conditions and released the loaded compounds in simulated intestinal fluid [9]. Cai et al. compared different de-esterification methods of low-methoxyl citrus pectin (LMP) and studied curcumin–pectin calcium gel beads, which exhibited controlled release and colon-targeted delivery potential [10]. Gugleva et al. developed a hybrid drug delivery platform with niosomal in situ gel for intravesical co-delivery of curcumin and gentamicin sulfate. This thermosensitive gel exhibited synergistic effect and controlled drug release (Figure 1) [65].

The development of curcumin delivery systems also involves analytical techniques for characterization and analysis. Ghodake et al. developed a stability-indicating HPTLC method for simultaneous determination of donepezil hydrochloride and curcumin in nasal gel, providing accurate results suitable for stability-indicating analysis [66]. Iwunze and McEwan explored the use of sol–gel-encapsulated curcumin as a sensor for small biomolecules, suggesting its potential for characterizing biomolecules [67]. Mohammad et al. developed an optical pH sensor using curcumin immobilized in sol–gel/chitosan–melamine hybrid matrices, demonstrating a linear response in the pH range of 9–13 with good reproducibility and fast response time [14].

### 2.1. Bioavailability Enhancement and Sustained Release of Curcumin in Food and Pharmaceutical Systems

Researchers have explored the use of protein-based gels as delivery vehicles for curcumin. Aliabbasi et al. developed an acid-induced cold-set gel using pinto bean protein isolate (PBI) and successfully embedded curcumin within the gel matrix. The gel exhibited sustained release of curcumin in simulated intestinal conditions, indicating its potential as a delivery system [68]. Du et al. utilized soybean protein isolate (SPI) to develop a low-oil-phase emulsion gel for curcumin delivery. The gel demonstrated excellent wettability and prevented oxidative deterioration of curcumin, making it suitable for protecting easily oxidized lipid-soluble nutrients in low-fat diets [69].

Gelation agents such as gellan gum (GG) and xanthan gum (XG) have also been investigated for their ability to improve the properties of curcumin-loaded gels. Du et al. studied the effect of GG on the gelation of yellow croaker roe protein isolate (pcRPI) and found that GG enhanced the water-holding capacity and microstructure of the gels. Moreover, GG effectively protected curcumin and slowed down its release in the gastrointestinal environment [70]. Geremias-Andrade et al. employed whey protein isolate and xanthan gum to produce curcumin-loaded emulsion-filled gels with improved color stability. The encapsulation of curcumin in solid lipid microparticles within the gels enhanced its stability [71].

In addition to protein-based gels, researchers have explored the use of Pickering emulsion gels as carriers for curcumin. Lei et al. developed Pickering emulsion gels stabilized by zein hydrolysate–chitin nanocrystal (CNC) coacervates. These emulsion gels exhibited improved rheological properties, emulsion stability, and antioxidant activity. The zein hydrolysate–chitin nanocrystals effectively enhanced the stability and controlled release of curcumin, increasing its bioaccessibility during digestion [72]. Lv et al. fabricated whey protein isolate (WPI) gel particles using high hydrostatic pressure (HHP) treatment and successfully formed Pickering emulsion gels. The resulting gels showed high loading efficiency of curcumin and excellent stability against light degradation [73]. Qiao et al. prepared Pickering emulsion gels stabilized by pea protein nanoparticles (PNPs) using heat-assisted pH shifting. The resulting emulsion gels exhibited improved curcumin bioaccessibility and stability. This research highlights the potential of this delivery system for enhancing the bioavailability of hydrophobic nutraceuticals such as curcumin [60].

Polysaccharide-based high-internal-phase emulsion (HIPE) gels have also been explored for curcumin delivery. Miao et al. formulated HIPE gels using sugar beet pectin, tannic acid, and chitosan complexes. These HIPEs exhibited a gel-like texture, improved stability, and enhanced curcumin bioaccessibility [74].

Additionally, Qazi et al. investigated the influence of gel structures on the gastric digestion and bioaccessibility of curcumin in dairy gels. The type of gel affected the release of curcumin-enriched oil and solubilized curcumin during gastric and intestinal digestion. This study provided insights for designing therapeutic dairy products that optimize the bioavailability of curcumin [59]. Pan et al. delved into the formation mechanism and curcumin bioaccessibility of emulsion gels based on sugar beet pectin (SBP). Emulsion gels stabilized by modified whey protein hydrolysates and SBP showed uniform networks and the highest curcumin bioaccessibility. These findings highlight the potential of emulsion gels for delivering curcumin and improving its bioavailability [58].

Furthermore, researchers have focused on developing oral delivery systems for curcumin. Tan et al. developed a food-grade oral delivery system using curcumin-loaded medium-chain triglycerides (MCTs)-encapsulated kappa-carrageenan (MCT-KC) gel beads. The MCT oil and kappa-carrageenan improved curcumin’s solubility, gastric resistance, and encapsulation efficiency. In vitro studies demonstrated the performance of the MCT-KC system, indicating its potential as a delivery carrier for hydrophobic compounds [75].

To maximize stability and minimize lipid oxidation, Vellido-Perez et al. designed an oil gelled-in-water emulsified curcumin delivery system. Through optimization of critical operating parameters, an optimal formulation was identified that achieved high curcumin load and minimal lipid oxidation [76].

### 2.2. Topical Delivery Systems for Inflammatory Conditions

Several studies have focused on developing optimized curcumin-based topical formulations. Abd El-Halim et al. developed a curcumin proniosomal gel with high entrapment efficiency and controlled release, showing promise for herpes simplex virus type 1 (HSV-1) infections [19]. Araujo et al. evaluated a curcumin transdermal gel for inflammatory bowel diseases, demonstrating a protective effect against oxidative stress [77]. Bakhshi et al. conducted a clinical trial comparing 1% curcumin nanomicelle gel and 2% curcumin gel for recurrent aphthous stomatitis (RAS), both showing efficacy in reducing pain and lesion size [20].

Transdermal gel formulations, such as those developed by Chaudhary et al., have been optimized using statistical design, emphasizing the importance of formulation optimization [21]. Innovative dermal delivery systems such as nanostructured lipid carriers (NLCs) and in situ gels have also been explored. Chen et al. developed curcumin-loaded NLCs and a thermosensitive in situ gel, demonstrating high entrapment efficiency and enhanced anti-inflammatory effects [22]. The use of two topical vehicle organogels and nanogels for curcumin delivery has shown potential, as seen in the study by Gonzalez-Ortega et al. [23].

Mucoadhesive gels loaded with curcumin solid lipid nanoparticles (CurSLNs) have shown promise in treating oral precancerous lesions. Hazzah et al. formulated a curcumin loaded CurSLN gel, demonstrating good mucoadhesion and complete healing in patients with oral erythroplakia [24]. For psoriasis treatment, various gel formulations have been developed. Iriventi et al. developed a nanosponge-based topical gel of curcumin and caffeine, showing sustained release and anti-psoriatic activity [25]. Jain et al. developed a liposphere gel formulation loaded with tacrolimus and curcumin, improving skin penetration and ameliorating psoriatic features [78]. Jain et al. investigated the synergistic potential of a nanostructured lipid carrier (NLC) gel of ibrutinib with curcumin, demonstrating enhanced drug flux and reductions in psoriasis severity score [79]. Combination formulations, such as the nanoemulsion gel containing curcumin, thymoquinone, and resveratrol developed by Khatoon et al., have also shown promise for psoriasis therapy [80].

To address challenges related to solubility and permeability, researchers have developed various delivery systems. Kumar et al. formulated a curcumin-loaded ethosomal gel for transdermal delivery, addressing solubility and permeability challenges [26]. Innovative gel formulations incorporating curcumin, such as the topical gel delivery system developed by Patel et al., have demonstrated enhanced skin permeation and anti-inflammatory effects [81]. Novel approaches involving complexation with beta-cyclodextrin have also been investigated, enhancing the solubility and permeability of curcumin [27].

Targeted delivery systems have been explored for rheumatoid arthritis treatment. Sana et al. developed curcumin-loaded transfersomes, exhibiting improved therapeutic efficacy and reduced pro-inflammatory cytokines [28]. Gel microemulsions have been investigated for topical cosmetic applications, showing potential for incorporating hydrophobic active ingredients such as curcumin [29]. Shehata et al. formulated curcumin into proniosome gels that can be hydrated into niosomal formulations, demonstrating enhanced skin permeability and significant anti-inflammatory activity [82].

Solid lipid nanoparticles (SLNs) have been studied for curcumin delivery in the treatment of pigmentation and irritant contact dermatitis (ICD), exhibiting controlled drug release and efficacy in reducing ICD-related symptoms [83]. Singh and Dabre formulated a controlled-release gel containing curcumin microspheres and diclofenac diethylamine for rheumatoid-arthritis-associated inflammation, showing improved anti-inflammatory efficacy [84]. To enhance solubility and transdermal delivery, Tripathi et al. utilized a hydrotropic solid-dispersion (HSD) approach with sodium salicylate, resulting in enhanced solubility and improved drug release [85]. Vila et al. developed a stable emulsion with curcumin and locust bean gum, demonstrating enhanced permeation of curcumin suitable for cutaneous applications [86].

Clinical studies have evaluated the efficacy of curcumin gel in the treatment of specific inflammatory conditions. Yaghoobi et al. conducted a study showing significant reduction in psoriasis symptoms with 1% curcumin gel compared to placebo [87].

### 2.3. Oral Health and Dental Applications of Curcumin

Curcumin has shown promising results in the treatment of recurrent aphthous stomatitis (RAS), a common oral condition characterized by painful ulcers. Deshmukh and Bagewadi compared the efficacy of curcumin gel with triamcinolone acetonide gel in treating RAS. Both gels demonstrated significant improvements in pain, size, and number of ulcers. Curcumin gel in particular exhibited strong antioxidant and anti-inflammatory properties, suggesting it as an effective alternative to steroids in RAS treatment [30].

In the context of oral leukoplakia, Fathima and Manoharan investigated the efficacy of curcumin oral gel compared to bleomycin. While bleomycin showed greater resolution of leukoplakic lesions, curcumin exhibited histopathological improvement of dysplasia [31]. These findings highlight the potential of curcumin as a therapeutic agent in managing precancerous oral lesions.

The development of effective drug delivery systems is crucial for enhancing the bioavailability and therapeutic efficacy of curcumin in oral applications. Fonseca-Santos et al. designed a mucoadhesive vehicle for buccal administration of curcumin to treat oral candidiasis. The formulation demonstrated mucoadhesive properties and ex vivo study showed enhanced curcumin retention in porcine esophageal mucosa. In vitro studies further revealed enhanced antifungal activity against Candida albicans [36]. This research highlights the potential of curcumin-based formulations in the management of oral fungal infections.

Furthermore, curcumin has been evaluated as a potential therapeutic agent in managing orthodontic-treatment-related inflammation. Samita et al. studied the effect of locally applied curcumin gel on myeloperoxidase (MPO) enzymatic activity in gingival crevicular fluid during orthodontic tooth movement. The gel significantly decreased MPO activity, indicating its potential anti-inflammatory effect in orthodontic treatment [88]. These findings suggest that curcumin may offer benefits in reducing inflammation associated with orthodontic procedures.

The antimicrobial properties of curcumin have also been explored in the context of root canal disinfection. Oda et al. evaluated the antimicrobial action of curcumin photoactivated by LED curing light in the presence of carbopol gel. The effectiveness of curcumin + LED curing light was comparable to methylene blue + diode laser, demonstrating its potential as an alternative disinfection method [32].

Moreover, curcumin has been investigated as an adjunctive therapy in the treatment of gingivitis. Pandey et al. evaluated the effects of oral curcumin gel as an adjunct to scaling and root planing (SRP) for treating gingivitis. The curcumin gel exhibited significant antiplaque and anti-inflammatory effects when used alongside SRP, improving clinical parameters [33]. These findings suggest the potential of curcumin as an adjunctive therapy for periodontal management.

### 2.4. Periodontal Therapy

Periodontal disease, a prevalent oral health condition characterized by inflammation and destruction of the supporting structures of the teeth, necessitates effective treatment strategies. While scaling and root planing (SRP) remain the conventional approach, researchers have been exploring adjunctive therapies to enhance treatment outcomes.

Anuradha et al. conducted a clinical study on curcumin gel as an adjunct to periodontal treatment, reporting significant reductions in plaque index, gingival index, and probing depth and a gain in clinical attachment level, signifying its potential effectiveness in periodontal therapy [89]. Moreover, Dave et al. found that topical curcumin gel combined with SRP resulted in greater reductions in plaque accumulation, sulcular bleeding, and pocket probing depth compared to SRP alone, thus emphasizing its effectiveness as an adjunct to periodontal treatment [90].

**Figure 2:**
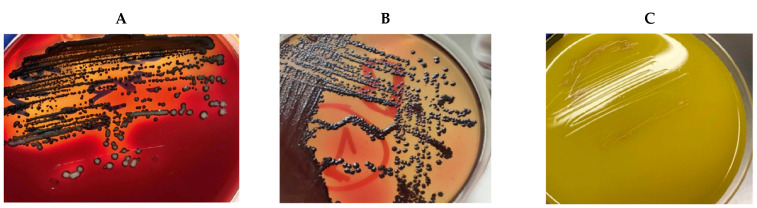
Pure isolated bacterial colonies of *P. gingivalis* after (**A**) 7 days and (**B**) 10 days on Columbia agar plate; (**C**) agar-formed media cultured from the tube with the MIC of curcumin showing no *P. gingivalis* growth [92].

In experimental periodontitis, Hosadurga et al. formulated a 2% curcumin gel and observed significant inhibition of edema and improvements in gingival index and probing pocket depth compared to in the control group, further highlighting its effectiveness in treating periodontitis [91]. Sha et al. demonstrated the antibacterial activity of curcumin against *P. gingivalis*, suggesting its utility in managing periodontal infections and promoting oral health (Figure 2) [92].

Comparative studies have been conducted to evaluate the efficacy of curcumin gel compared to other adjunctive treatments. Hugar et al. compared the efficacy of curcumin gel and chlorhexidine gel as adjuncts to SRP in periodontal therapy and found that curcumin gel demonstrated greater reduction in periodontal parameters, establishing its superiority as an adjunct to SRP [93]. Similarly, Ravishankar et al. evaluated the effect of curcumin gel compared to ornidazole gel in treating chronic periodontitis and reported significant improvements in pocket probing depth, plaque index, and clinical attachment loss with curcumin gel, indicating its potential as an adjunct to nonsurgical periodontal therapy [94].

Furthermore, curcumin gel has shown promising results in managing specific conditions. It was evaluated as an adjunct to scaling and root planing (SRP) in the treatment of chronic periodontitis by Kaur et al., who reported a mild adjunctive benefit in reducing gingival inflammation [95]. Additionally, Meghana et al. compared the efficacy of curcumin gel and noneugenol periodontal dressing following periodontal flap surgery, and both treatments effectively reduced tissue edema, enhanced wound healing, and reduced pain perception [96].

Curcumin gel has also been investigated for its impact on systemic factors and its potential synergistic effects with existing therapies. Mohammad evaluated the effect of curcumin gel on serum micronutrients and pro-inflammatory cytokines in chronic periodontitis patients and found significant reductions in clinical parameters, inflammatory mediators, and copper levels, while zinc and magnesium levels increased [97]. Moreover, in experimental periodontitis, Mohammad et al. demonstrated that curcumin gel effectively reduced inflammation, bone resorption, and osteoclast numbers, indicating its potential osteogenesis and healing effects [98]. In a study by Mohammad et al., the antioxidant effects of curcumin gel in diabetes-induced periodontitis were evaluated, revealing a significant reduction in oxidative stress and increased antioxidant enzyme levels [99].

### 2.5. Oral Submucous Fibrosis (OSMF) Management

Oral submucous fibrosis (OSMF) is a chronic, progressive, and potentially malignant disorder characterized by fibrosis of the oral submucosal tissues. It is associated with various symptoms such as burning sensation and restricted mouth opening, leading to significant morbidity and impaired quality of life for affected individuals. Current treatment options for OSMF are limited and often associated with side effects. Therefore, there is a growing interest in exploring natural compounds with therapeutic potential for managing OSMF.

Chandrashekar et al. conducted a study to compare the efficacy of topical curcumin gel and buccal mucoadhesive patches for OSMF. Both formulations demonstrated significant improvements in reducing burning sensation, increasing mouth opening, and reducing serum lactate dehydrogenase levels. These findings indicate the potential of curcumin-based formulations for the treatment of OSMF [37].

In a study by Lanjekar et al., the efficacy of curcumin gel, triamcinolone–hyaluronidase gel, and their combination was evaluated for OSMF treatment. The combination therapy showed the greatest improvement in mouth opening, while the triamcinolone–hyaluronidase group reported reduced burning sensation. This study highlights the therapeutic effects of curcumin on OSMF and demonstrated that combination therapy enhances its utilization and drug delivery [38].

Furthermore, Nerkar Rajbhoj et al. compared the efficacy of curcumin gel and aloe vera gel in managing OSMF. Both gels exhibited improvements in mouth opening; however, aloe vera gel provided better relief from burning sensation, making it a more effective option without notable side effects [39].

### 2.6. Gastrointestinal Applications of Curcumin

Researchers have been exploring various delivery systems and formulations to optimize the bioavailability and therapeutic efficacy of curcumin. In this section, we discuss several studies that investigated different approaches for curcumin delivery in the gastrointestinal tract.

Araiza-Calahorra et al. conducted a study on Pickering emulsions stabilized by colloidal gel particles for curcumin delivery. Their modified emulsions demonstrated improved stability against coalescence and enhanced cellular uptake of curcumin. This research suggests the potential of utilizing Pickering emulsions as a promising delivery system for lipophilic bioactive compounds, including curcumin [40].

Another study by Fontes-Candia et al. focused on agar and kappa-carrageenan emulsion gels and oil-filled aerogels as curcumin carriers. The researchers observed distinct behavior and structural changes of the gels during in vitro digestion influenced by the presence of curcumin. This investigation provides valuable insights into the interaction between curcumin and emulsion gels, shedding light on their potential application in gastrointestinal delivery systems [41].

Kathpalia et al. formulated a gastroretentive floating in situ gelling system of solubility-enhanced curcumin specifically designed for gastric ulcers. This system exhibited extended release of curcumin for up to 12 h, indicating its potential as a reliable delivery platform for curcumin in treating gastric ulcers [42]. Similarly, Padhan et al. developed a gastroretentive in situ gelling system of curcumin for gastric ulcers associated with Helicobacter pylori. Their curcumin in situ gel demonstrated suitable properties such as floating lag time, duration, and controlled drug release, which could be regulated by the concentration of sodium alginate [43].

In another approach, Su et al. fabricated whey protein isolate (WPI)/kappa-carrageenan (kappa-CG) composite emulsion gels using ultra-high-pressure (UHP) technology for curcumin delivery. This study investigated the physical properties and release behavior of the emulsion gels, examining different formulations and processing conditions. It also evaluated the release of curcumin during simulated gastrointestinal digestion, providing valuable insights into the potential of WPI/kappa-CG emulsion gels as an effective curcumin delivery system [44].

### 2.7. Wound Healing

One approach to harnessing the wound healing properties of curcumin is through the development of novel delivery systems. Aghamoosa et al. developed a silver–curcumin nanogel that exhibited desirable properties and demonstrated efficacy in promoting wound healing in a burn model [45]. Similarly, Das et al. prepared a composite gel containing gelatin, F127, and lecithin for enhanced wound healing which facilitated the controlled release of curcumin and improved wound healing in a murine model [46]. These studies highlight the potential of curcumin-based formulations as effective tools for wound management.

**Figure 3:**
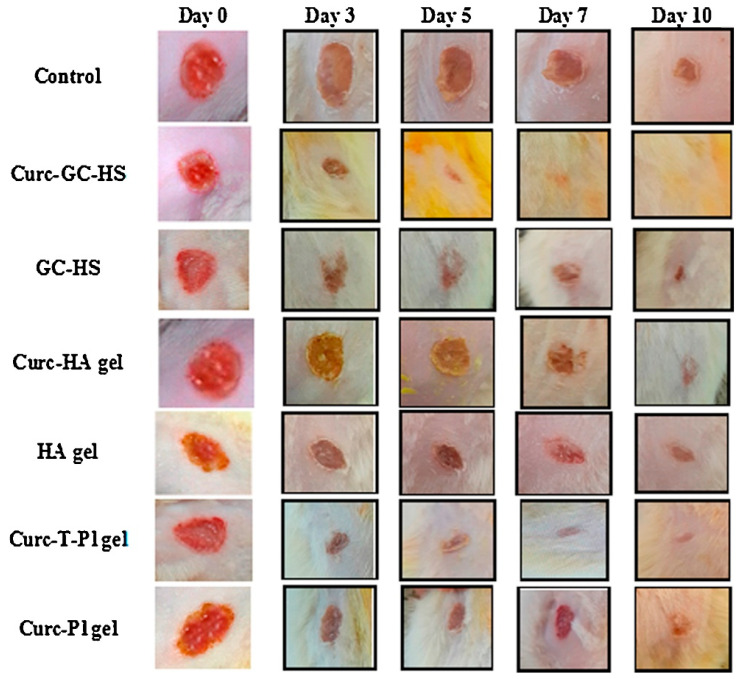
Stages of burn wound healing potential study of curcumin-loaded novel self-assembled nanogels gel–core hyaluosome (Curc-GC-HS) compared to conventional transfersomal gel (Curc-T-Pl gel) and other conventional gels Curc–HA gel, Curc in pluronic gel (Curc–Pl gel), gel-core hyaluosome (GC-HS), and HA gel. Adapted with permission [47].

The use of nanogels as delivery vehicles for curcumin has garnered attention in wound healing research. El-Refaie et al. evaluated curcumin-loaded gel–core hyaluosomes (GC-HS) and demonstrated their high entrapment efficiency, bilayer structure, and improved healing rate with reduced scar formation in vivo (Figure 3) [47]. Guo et al. investigated the therapeutic effect of curcumin-loaded self-microemulsifying gel and found that it exhibited higher skin flux and enhanced wound healing compared to commercial gels [48]. These studies underline the potential of nanogel-based systems in facilitating efficient delivery of curcumin to promote wound healing.

Furthermore, the development of three-dimensional (3D) scaffolds incorporating curcumin has shown promise in wound healing applications. Karahaliloglu demonstrated the effectiveness of an electroresponsive silk fibroin (SF) hydrogel loaded with curcumin as a 3D scaffold for wound healing which exhibited reduced bacteria and no toxicity to healthy cells [100]. This approach offers a versatile platform for delivering curcumin to wound sites and promoting tissue regeneration.

In addition to its individual effects, curcumin has been investigated in combination with other compounds to enhance its wound healing properties. Khan et al. formulated a gel containing allicin and curcumin which exhibited superior wound healing activity compared to conventional treatments [101]. These findings highlight the potential synergistic effects of curcumin in combination with other bioactive compounds for improved wound management.

The wound healing potential of curcumin has also been explored in various contexts. Kim et al. investigated the wound healing effect of transdermal curcumin gel, which exhibited antioxidant activity, inhibition of nitric oxide production, and promotion of wound healing in vivo [102]. Raman and Pitty conducted a study comparing the effectiveness of topical curcumin gel and triamcinolone acetonide oral paste in reducing pain and size of recurrent minor oral aphthous ulcers, demonstrating the efficacy of curcumin gel in wound healing applications [51]. These studies emphasize the diverse applications of curcumin in wound management.

Moreover, advancements in formulation techniques have facilitated the development of curcumin-based delivery systems with improved efficacy. Kotian et al. prepared curcumin nanocrystals and incorporated them into a gel, resulting in enhanced drug release, skin permeation, and wound healing activity compared to unprocessed curcumin without causing skin irritation [49]. Pisitsak and Ruktanonchai embedded curcumin in a sol–gel coating on cotton textiles, which exhibited controlled release and antibacterial properties, suggesting their potential as wound dressing materials [50]. These studies highlight the importance of formulation strategies in maximizing the therapeutic potential of curcumin for wound healing applications.

### 2.8. Ophthalmic Delivery

The use of curcumin in ophthalmic drug delivery has emerged as a promising strategy to overcome the limitations of conventional eye drops and enhance the therapeutic efficacy of the drug. Several innovative formulations have been developed to improve the delivery and release of curcumin to the ocular surface and its permeation.

**Figure 4:**
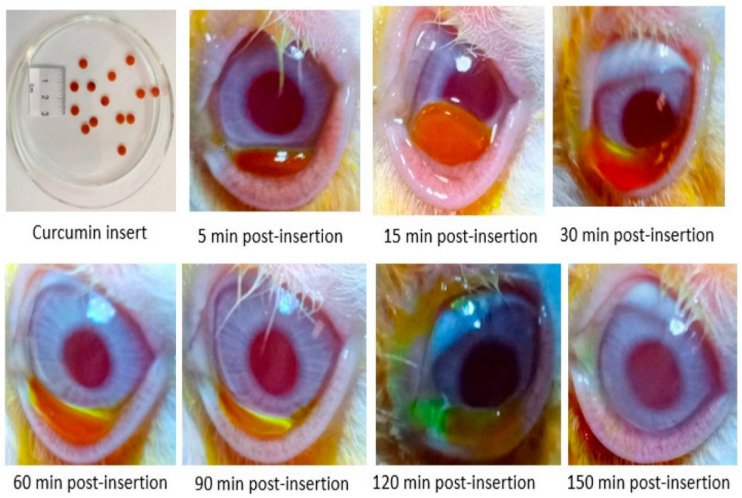
Image of curcumin in situ gelling polymeric solid insert into the inferior conjunctival cul de sac of rabbit eye, transformed from solid to gel for enhancing ocular performance [52].

Abdelkader et al. investigated the use of in situ gelling polymeric inserts for curcumin delivery to the ocular surface. Their study demonstrated that these inserts exhibited improved characteristics, release, and permeation compared to a traditional suspension. Furthermore, the inserts were well tolerated and provided sustained release of curcumin. This research suggests that in situ gelling polymeric inserts could serve as a viable alternative to conventional eye drops for efficient curcumin delivery (Figure 4) [52].

Duan et al. focused on the development of an ion-sensitive mixed micelle in situ gel system for ophthalmic delivery of curcumin. Their system demonstrated sustained release properties and higher corneal permeation when compared to a curcumin solution. These findings highlight the potential of the ion-sensitive mixed micelle in situ gel system as an effective carrier for curcumin delivery to the eye [53].

In a similar vein, Sai et al. developed a mixed micelle in situ gelling system of curcumin for ophthalmic drug delivery. This innovative system not only improved the solubility and corneal penetration of curcumin but also exhibited a longer retention time on the corneal surface. The prolonged retention time is crucial for enhancing drug bioavailability and therapeutic efficacy. The study concluded that the mixed micelle in situ gelling system holds promise as an effective option for ophthalmic drug therapy utilizing curcumin [103].

### 2.9. Other Applications of Curcumin

In addition to ophthalmic drug delivery, curcumin gels have shown promise in several other therapeutic applications, ranging from food products to corrosion protection, protein staining, contraception, and nutraceutical delivery.

Brito-Oliveira et al. focused on developing emulsion-filled gels using soy protein isolate and xanthan gum as the gel matrix. The incorporation of curcumin-loaded solid lipid microparticles in these gels enhanced their mechanical properties and stability. This research suggests that curcumin encapsulation in gels could serve as an alternative to artificial dyes in gelled food products, providing a natural and functional ingredient [54].

Asefi et al. investigated the effect of curcumin on orthodontic tooth movement (OTM) and found that it inhibited root and bone resorption, osteoclastic recruitment, and angiogenesis, suggesting its potential benefits in preventing side effects associated with orthodontic treatment [104].

Gaurav et al. aimed to develop a safe and effective vaginal contraceptive using a copper–curcumin complex incorporated into a cyclodextrin inclusion complex. The vaginal gel demonstrated excellent spermicidal activity and safety in preclinical studies. This novel contraceptive platform utilizing curcumin offers a promising approach for contraception [52].

Ishak et al. investigated the incorporation of curcumin extracts into sol–gel coatings for corrosion protection of mild steel. The resulting coatings exhibited high inhibition efficiency and hydrophobic properties, indicating their potential for corrosion mitigation. This research highlights the potential of curcumin-based sol–gel coatings in the field of corrosion protection [56].

Kurien et al. explored the utility of curcumin as a protein stain gel. They demonstrated that heat-solubilized curcumin could effectively stain proteins in a manner comparable to Coomassie brilliant blue (CBB) at a significantly lower cost and without requiring destaining. This cost-effective and environmentally friendly alternative offers curcumin as an ideal protein stain [57]. Furthermore, Kurien et al. solubilized curcumin in Tween 80 or Triton X-100, creating an efficient protein stain gel. The solubilized curcumin exhibited high solubility, efficient staining, and stability without the need for destaining, providing a nontoxic and environmentally friendly alternative to traditional protein stains [105].

Raduly et al. focused on coating cotton fabrics with multifunctional nanosols generated through sol–gel reactions. The resulting coatings exhibited hydrophobicity, fluorescent and antimicrobial properties, as well as pH-dependent color change. These multifunctional coatings retained their properties even after washing cycles, offering potential applications in textiles requiring signaling, self-cleaning, or antibacterial properties [106].

Scomoroscenco et al. investigated the stability and antioxidant properties of curcumin encapsulated in microemulsion and gel microemulsion systems. These systems effectively protected curcumin from degradation and demonstrated a synergistic effect between curcumin and vegetable oil in terms of antioxidant capacity. This research highlights the potential of curcumin-based microemulsion and gel microemulsion systems as stable and effective antioxidant formulations [61].

Wang et al. developed an ion-sensitive in situ gelling system for the intranasal administration of curcumin. The system, based on a microemulsion formulation, demonstrated desirable properties and enhanced the bioavailability of curcumin through intranasal administration. Additionally, the system exhibited potential brain-targeting properties, making it a promising approach for delivering curcumin to the central nervous system [62].

Table 1 summarizes recent research on the preparation of therapeutic curcumin gel formulations and delivery systems.

## 3. Concluding Remarks

The field of research on curcumin gel compositions for therapeutic purposes has seen significant advancements and promising developments. Various innovative approaches have been explored to enhance curcumin’s delivery, stability, dissolution characteristics, and targeted release [5,6,7,8,9,10,11,15,16,75,76,108,109]. These formulations offer controlled release properties, improved bioavailability, enhanced therapeutic benefits, and targeted delivery for specific applications such as Alzheimer’s treatment, gastrointestinal stability, controlled drug release, and intravaginal delivery.

In food and pharmaceutical systems, the focus has been on enhancing bioavailability and sustaining curcumin release [68,69,70,71,72,73,74,75,76]. These advancements have contributed to the development of functional food ingredients and delivery systems, offering improved oxidative stability, enhanced antioxidant properties, and reduced lipid content.

For inflammatory conditions, researchers have explored topical delivery systems [19,20,21,23,25,26,28,77,78,79,81,82,83,84,85,86,87]. These approaches provide potential anti-inflammatory activity, improved efficacy, permeation, stability, and targeted delivery, showing promise for managing inflammatory conditions such as rheumatoid arthritis and psoriasis.

Curcumin has demonstrated its potential in various oral health applications [30,31,32,33,36,88,115]. It has been investigated for the treatment of minor recurrent aphthous stomatitis (RAS) and oral leukoplakia and as an adjunct in endodontic therapy, gingivitis treatment, and oral wound healing. Additionally, curcumin gel shows potential as an adjunctive therapy in periodontal treatment [92,93,94,96,97,99,104,116,117,118], reducing bone and root resorption during orthodontic treatment and enhancing the efficacy of scaling and root planing (SRP) in treating chronic periodontitis.

Moreover, curcumin gel and mucoadhesive patches show potential as noninvasive management options for oral submucous fibrosis (OSMF) [37,38,39], offering symptom relief, improved oral function, and enhanced drug delivery. Complementary treatments using curcumin alongside other compounds have shown effectiveness in managing OSMF symptoms.

In gastrointestinal applications, curcumin’s potential lies in enhancing bioavailability and cellular uptake [40,41,42,43,44]. Various formulations offer extended-release properties, gastric floatability, and sustained drug release, contributing to improved therapeutic outcomes.

The research has also highlighted curcumin’s promise in wound healing applications [45,46,47,48,49,50,51,100,101,102,119]. Various formulations have shown positive effects in wound healing, making curcumin gel a valuable option for managing oral aphthous ulcers.

Furthermore, curcumin shows potential in ophthalmic drug delivery [52,53,103], with polymeric in situ gelling inserts and ion-sensitive mixed micelle in situ gel systems offering improved properties for ocular therapy.

Additionally, curcumin has demonstrated its versatility beyond traditional therapeutic uses [54,55,56,57,61,62,106], finding applications as a natural food dye alternative, a topical contraception option, a corrosion inhibitor, a reversible protein stain, and more. These findings highlight the diverse applications and potential benefits of curcumin in various fields, including food, contraception, corrosion protection, and drug delivery.

Despite its numerous therapeutic benefits, researchers have also noted potential safety concerns related to curcumin’s genotoxic properties [122], warranting further investigation in this area.

## 4. Conclusions

Curcumin holds great promise in advancing various fields of research and therapeutic applications. Significant advancements have been made in terms of its physicochemical properties and drug delivery systems, enhancing curcumin’s delivery, stability, and targeted release for specific applications. Bioavailability enhancement and sustained release strategies have been developed for food and pharmaceutical systems, improving curcumin’s stability, antioxidant properties, and controlled release properties. Topical delivery systems show potential for managing inflammatory conditions, while curcumin has shown promise in various oral health applications, periodontal therapy, and the management of oral submucous fibrosis. Gastrointestinal applications, wound healing interventions, ophthalmic drug delivery, and diverse therapeutic applications have further demonstrated the potential of curcumin. However, it is important to consider the safety concerns associated with curcumin, as reports suggest its genotoxic potential and DNA-damaging effects. Continued research and development in these areas are crucial to fully understand curcumin’s therapeutic potential, optimize its delivery systems, and ensure its safe and effective use in various biomedical and pharmaceutical applications.

## Figures and Tables

**Table 1:** A summary of curcumin gel compositions and their use in various areas.

Curcumin Delivery Systems		
Materials and Methods	Applications and Advancements	Ref.
Curcumin-loaded solid lipid nanoparticles (SLN) in thermoresponsive gel	Enhanced physicochemical properties, sustained release, potential for Alzheimer’s disease treatment	[6]
Curcumin–soybean phosphatidylcholine (CUR-SPC)-complex-loaded NPs in HA gel	Prevention of postoperative peritoneal adhesion, harnessing advantages of CUR, SPC, and HA	[4]
Curcumin loaded whey protein aggregates/k-carrageenan hydrogels	Potential delivery system, protecting curcumin during digestion, enabling targeted delivery to the colon	[5]
Curcumin phytosomal soft gel formulations using different vehicles	Enhancing bioavailability, overcoming solubility limitations, improved delivery of curcumin	[7]
Curcumin encapsulated in surface-modified MIL-100(Fe) with CMC	pH-responsive drug delivery, controlling release profile of hydrophobic drugs, biomedical applications	[8]
Starch-based emulsion gel beads prepared by inverse emulsion gelation	Ideal carriers for loading hydrophilic and hydrophobic ingredients, controlled release systems	[9]
Low-methoxyl-citrus-pectin-based curcumin–pectin calcium gel beads	Potential in functional foods and drug delivery, colon-targeted delivery, novel delivery systems	[10]
Stability-indicating HPTLC method for simultaneous determination of donepezil hydrochloride and curcumin in nasal gel	Reliable analytical tool for quality control and stability testing in nasal gel formulation	[66]
Study on dissolution, gel properties, and characterization of amorphous curcumin	Understanding gelation potential of amorphous materials, improving drug delivery systems	[107]
Sol–gel-encapsulated curcumin	Potential sensor for biomolecules, contributes to sensor technologies	[67]
Curcumin in kappa-carrageenan gel with metal chlorides	Prolonging antioxidant and anti-inflammatory effects, benefiting healthcare	[108]
Polyphenon 60 (P60) + curcumin (CUR) nanoemulsion gel	Combined antibacterial treatment for E. coli infections, enhanced drug availability	[109]
Nanostructured lipid carriers (NLCs)-loaded chitosan/carbopol hybrid gel	Promising delivery system for malarial parasite management	[17]
Curcumin-loaded poly(2-hydroxyethyl methacrylate) nanoparticles	Therapeutic potential in ovarian cancer treatment	[11]
pH-sensitive curcumin-loaded microemulsion gel system	Smart drug delivery system with enhanced controlled release	[110]
Hydrogel carrier of oligo-conjugated linoleic acid vesicles	Injectable/3D printable drug delivery system with antioxidant property	[12]
Albumin nanoparticles loaded with curcumin and thermoresponsive gels	Potential for ocular drug delivery with enhanced bioavailability	[13]
Curcumin reagent immobilized in sol–gel/chitosan–melamine hybrid matrices	Optical pH sensor for pH measurement	[14]
Cryogel-based encapsulation of curcumin in chitosan, kappa-carrageenan, and NaCMC	Advancements in controlled release technology	[15]
Film-forming nanogels containing poly(lactide-co-glycolide) and gelatine nanoparticles	Improved permeability and sustained diffusion	[16]
Preparation of a hydrotropic solid dispersion (HSD) loaded with curcumin	Enhancing topical delivery and potential for transdermal drug delivery	[85]
Development of a microemulsion-based in situ ion-sensitive gelling system	Enhanced curcumin absorption and brain targeting, intranasal drug delivery	[62]
Preparation of Ca2+-induced emulsion gels using HHP–pectin and erythritol	Low-calorie food options and delivery of fat-soluble nutrients	[111]
Curcumin-loaded liposomal solid gels prepared with freeze-drying technique	Enhanced therapeutic efficacy against hepatitis C virus	[18]
Niosomal in situ gel system prepared by thin-film hydration method	Treating urinary bladder-related conditions with sustained release	[65]
**Bioavailability Enhancement and Sustained Release of Curcumin in Food and Pharmaceutical Systems**		
Acid-induced cold-set gels based on pinto bean protein isolate (PBI) with curcumin	Sustained and controlled release of curcumin, enhancing bioavailability	[68]
Low-oil-phase emulsion gel prepared via high-pressure homogenization of SPI-Cur-NPs	Protection of oxidized lipid-soluble nutrients, advancements in antioxidant-rich food	[69]
Binary gels loaded with curcumin formed from large yellow croaker roe protein isolate and gellan gum	Improved water-holding ability, controlled release properties, versatile applications	[70]
Curcumin-loaded solid lipid microparticles incorporated in whey protein isolate and xanthan gum gels	Reducing lipid content in food, improving color stability while maintaining texture	[71]
Pickering emulsion gels stabilized by zein hydrolysate–chitin nanocrystals coacervates	Enhanced stability, delayed lipolysis, sustained nutrient release, applications in food/pharma	[72]
Whey protein isolate (WPI) gel particles with curcumin	Food-grade stabilizers for Pickering emulsions, enhanced stability against degradation	[73]
Polysaccharide-based high-internal-phase emulsion gels (HIPEs) with SBP, TA, and CS + curcumin	Soft solids with natural ingredients, stability enhancement, delayed lipid digestion	[74]
Emulsion gels with modified whey protein hydrolysates and curcumin	Delivery systems enhancing curcumin bioavailability	[58]
Curcumin nanoemulsion-containing dairy gels	Understanding gel structure impact on curcumin bioavailability	[59]
Pea protein nanoparticles (PNPs) and curcumin	Stabilizing Pickering emulsion gels for enhancing hydrophobic nutraceutical bioavailability	[60]
Curcumin-loaded MCT-KC gel bead formulation	Natural and food-grade carrier for enhancing hydrophobic compound delivery	[75]
Designing an oil gelled-in-water curcumin-loaded emulsion	Rational design for stabilizing and delivering bioactive compounds	[76]
**Topical Delivery Systems for Inflammatory Conditions**		
Curcumin proniosomal gel	Effective topical delivery system for HSV-1 treatment, insights into molecular interactions	[19]
Curcumin transdermal gel with Pentravan^®^	Reducing oxidative stress and inflammation in inflammatory bowel diseases	[77]
Efficacy of 1% curcumin nanomicelle gel and 2% curcumin gel	Enhanced healing of recurrent aphthous stomatitis, improved treatment outcomes	[20]
Gel formulations of diclofenac diethylamine (DDEA) and curcumin (CRM)	Optimized gel formulations for transdermal delivery, advancements in formulation	[21]
Curcumin-loaded nanostructured lipid-carriers-based thermosensitive in situ gel	Efficient dermal delivery system, improved permeation, anti-inflammatory effects	[22]
Curcumin-loaded gel carriers (organogels and O/W-type nanogels)	Improved topical formulations for inflammatory conditions	[23]
Curcumin-loaded solid lipid nanoparticles (CurSLNs) in mucoadhesive gel matrix	Local treatment of precancerous lesions with reduced side effects	[24]
Nanosponge with beta-CD and curcumin-loaded topical gel	More effective anti-psoriatic therapy, advancing psoriasis treatment	[25]
Liposphere gel formulation of tacrolimus and curcumin	Treatment strategy for psoriasis, addressing solubility and skin penetration	[78]
Ibrutinib and curcumin-loaded nanostructured lipid carrier (NLC)	Management of psoriasis, integrated benefits in reducing inflammation	[79]
Silica-based formulations with glycyrrhizic acid and curcumin	Superior treatment option for psoriasis, enhancing anti-psoriatic efficacy	[112]
Nanoemulsion gel formulation containing curcumin, resveratrol, and thymoquinone	Promising approach for psoriasis therapy, overcoming solubility and permeation issues	[80]
Curcumin-loaded ethosomal gel prepared by ethanol and soya lecithin	Potential solution for transdermal delivery, overcoming solubility and permeability challenges	[26]
Curcumin gel with Carbopol 934P and hydroxypropyl cellulose	Topical application with anti-inflammatory effects, alternative treatment option	[81]
Tailored water-in-oil emulsion systems with polyglycerol polyricinoleate (PGPR) and curcumin	Effective delivery of curcumin for various applications, controlled release platform	[113]
Curcumin–beta-cyclodextrin nanoparticle complex (BCD-CUR-N)	Improved solubility and skin penetration, potential for transdermal delivery	[27]
Curcumin-loaded transferosomes (Cur-TF) embedded in Carbopol 934 gel	Targeted topical treatment of rheumatoid arthritis, improved therapeutic efficacy	[28]
Gel microemulsions with grape seed oil and curcumin	Effective systems for curcumin delivery in dermatocosmetics	[29]
Curcumin-loaded niosomal emulgel with nonionic surfactants	Efficient carrier for transdermal drug delivery, enhanced anti-inflammatory effect	[82]
Curcumin solid lipid nanoparticles (CUR-SLNs) in carbopol gel	Safe and effective alternative for irritant contact dermatitis treatment	[83]
Curcumin microspheres in gel formulation	Potential treatment option for arthritis inflammation	[84]
Hydrotropic solid dispersion (HSD) in curcumin gel	Greener approach for enhancing topical delivery of poorly soluble compounds	[85]
Curcumin with choline and geranic acid ionic liquid (CAGE-IL)	Potential for transdermal drug delivery and cutaneous drug delivery systems	[86]
1% curcumin gel	Modest but continuous effect on plaque psoriasis	[87]
Curcumin-loaded water-responsive gel (CUR-WRG)	Enhanced efficacy of topical drug delivery against psoriasis	[114]
**Oral Health and Dental Applications of Curcumin**		
Curcumin gel	Safer and accessible therapeutic option for minor recurrent aphthous stomatitis (RAS)	[30]
Topical bleomycin and curcumin oral gel with antioxidants	Potential in treating oral leukoplakia	[31]
Mucoadhesive vehicle formulation of PPG-5-CETETH-20 and oleic acid	Effective treatment for oral candidiasis	[36]
Clinical trial comparing amlexanox paste and curcumin oral gel	Safe alternative for managing RAS minor	[115]
Curcumin + carbopol gel + LED curing light	Alternative to conventional photodynamic therapy for disinfecting root canals	[32]
Randomized clinical trial evaluating the effectiveness of curcumin gel	Potential alternative for managing gingival diseases	[33]
Clinical study evaluating locally applied 1% curcumin gel during orthodontic treatment	Implications for managing inflammation during orthodontic tooth movement	[88]
**Periodontal Therapy**		
Adjunctive use of curcumin gel with scaling and root planning (SRP)	Enhancing outcomes of periodontal therapy, anti-inflammatory properties	[89]
Adjunctive use of curcumin gel with SRP	Improved outcomes in chronic periodontitis, potential complementary treatment	[90]
Adjunctive use of curcumin oral gel	Effective improvement in periodontal pocket parameters	[34]
2% curcumin gel	Effective treatment of experimental periodontitis	[91]
Comparison of chlorhexidine gel and curcumin gel	Both gels as adjuncts to scaling and root planing, curcumin as an alternative	[93]
Curcumin gel as an adjunct to scaling and root planing	Limited additional benefits in reducing gingival inflammation	[95]
Curcumin gel and noneugenol periodontal dressing for post-surgery treatment	Effectiveness in reducing tissue edema and promoting wound healing	[96]
Curcumin gel injection with scaling and root planing	Reduction of clinical parameters and inflammatory mediators	[97]
Scaling and root planing combined with curcumin	Comparable results to tetracycline in reducing inflammation and bone resorption	[98]
Curcumin gel as an adjunct to SRP in diabetes-induced periodontitis	Reducing oxidative stress and improving antioxidant enzyme levels	[99]
In situ gel formulation of curcumin	Effective treatment of periodontal pockets	[35]
Curcumin + carbopol gel + LED curing light	Alternative to conventional photodynamic therapy for root canal disinfection	[32]
Split-mouth design study with curcumin gel	Potential adjunct to nonsurgical periodontal therapy	[94]
Evaluation of curcumin’s antibacterial activity against *P. gingivalis*	Effective antibacterial action at low concentrations	[92]
Curcumin gel in experimental periodontitis using Wistar rats	Effective in treating experimental periodontitis and preventing bone destruction	[116]
Clinical trial evaluating subgingival delivery of curcumin gel as an adjunct to SRP	Effective anti-inflammatory and antibacterial properties, alternative to chlorhexidine	[117]
Clinical trial comparing scaling and root planing alone, curcumin application, and curcumin photodynamic therapy	Curcumin photodynamic therapy as an adjunct to scaling and root planing improves treatment outcomes	[118]
**Oral Submucous Fibrosis (OSMF) Management**		
Curcumin gel and buccal mucoadhesive patches	Noninvasive and effective treatment for oral submucous fibrosis (OSMF)	[37]
Curcumin mucoadhesive semisolid gel and combination gels	Therapeutic effects in treating OSMF, potential role in treating oral cancers and precancerous lesions	[38]
Randomized clinical trial comparing curcumin gel and aloe vera gel	Efficacy in managing OSMF symptoms, aloe vera gel as adjuvant treatment	[39]
**Gastrointestinal Applications of Curcumin**		
Pickering emulsions with whey protein nanogel and whey protein isolate + dextran conjugate microgel	Enhanced delivery of curcumin, potential for functional foods	[40]
Emulsion gels with agar, kappa-carrageenan, and oil-filled aerogels + curcumin	Effective carriers with controlled release for gastrointestinal applications	[41]
Dry suspension floating in situ gelling system of curcumin	Locally treating gastric ulcers, controlled drug release in gastric environment	[42]
Gastroretentive floating in situ gelling system with curcumin	Extended gastric retention for improved therapeutic effectiveness	[43]
Composite emulsion gels with whey protein isolate/kappa-carrageenan	Controlled release and improved stability, gastrointestinal fate study	[44]
**Wound Healing**		
Silver–curcumin nanoparticles prepared by high-pressure homogenization	Enhanced wound healing and potential for burn treatment	[45]
Composite gel prepared from gelatin, F127, and lecithin	Promising wound healing approach with improved properties and bioavailable curcumin release	[46]
Curcumin gel-core hyaluosome (Curc-GC-HS) nanogels	Enhanced curcumin skin penetration, wound healing promotion, potential for skin cancer therapy	[47]
Curcumin-loaded self-microemulsifying gel (olive oil:glycerol = 1:2, PBS, and surfactant RH40)	Advanced tool for wound healing, controlled and sustained drug delivery	[48]
Curcumin-loaded silk fibroin e-gel scaffolds	Improving wound healing activity and serving as a wound dressing material	[100]
Allicin and curcumin gel using Carbopol 940	Excellent wound healing activity, alternative for wound care	[101]
Curcumin gel prepared with Carbopol 934 and propylene glycol	Promotes wound healing through transdermal delivery of curcumin	[102]
Curcumin nanocrystal-based gel prepared by sonoprecipitation	Enhances wound healing efficacy of curcumin	[49]
Coating cotton textiles with acid-catalyzed silica xerogel	Potential as wound dressing materials with prolonged curcumin release	[50]
Single-blinded randomized study on topical 2% curcumin gel	Effective reduction of pain and ulcer size in oral aphthous ulcers	[51]
Curcumin-loaded chitosan-based nanoemulsion gel	Enhanced skin deposition and retention for effective wound healing	[119]
**Ophthalmic Delivery**		
Polymeric inserts for ocular delivery of curcumin	Improved mechanical properties, sustained release for ophthalmic drug delivery	[52]
Curcumin-loaded ion-sensitive mixed micelle in situ gel (Cur-MM-ISGs)	Prolonged ocular retention, improved permeability, sustained release	[53]
Curcumin mixed micelles (Cur-MMs) in gellan gum gels	Advances ocular drug delivery, addressing curcumin’s solubility challenges	[103]
**Other Therapeutic Applications of Curcumin**		
Synthesis of solid-state fluorescent selenium quantum dots by solvothermal-assisted sol–gel method	Curcumin sensing applications, advancements in fluorescence-based sensing techniques	[120]
Copper–curcumin complex with beta-cyclodextrin	Novel metal–herbal approach for vaginal delivery, safe and effective topical contraception	[55]
Curcumin-incorporated hybrid sol–gel	Potential applications in corrosion protection for mild steel in acidic environments	[56]
Curcumin solubilization in water with heat/pressure treatment and detergents	Efficient, nontoxic, and environmentally friendly protein stain, increased bioavailability with Tween 80	[105]
Fabrication of multifunctional coatings on cotton fabrics using nanosols and curcumin derivatives	Coated cotton fabrics exhibit signaling, self-cleaning, and antibacterial properties	[106]
Encapsulation of curcumin in O/W microemulsion systems and gel microemulsions	Stable products with enhanced antioxidant activity for pharmaceuticals and cosmetics	[61]
Non-aqueous gel and curcumin analogue-loaded nanoparticle gel formulations	Topical formulations with potential applications and antioxidant activity	[121]
Curcumin gel prepared in gelatin hydrogel and chitosan hydrogel	Adjunctive treatment to preserve tooth and bone integrity during orthodontic treatment	[104]
Genotoxicity of curcumin in human lymphocytes	Highlights potential genotoxicity in human lymphocytes, warrants further investigation	[122]

## Data Availability

Not applicable.
